# Impact of Gastrointestinal Digestion on the Anti-Inflammatory Properties of Phlorotannins from *Himanthalia elongata*

**DOI:** 10.3390/antiox11081518

**Published:** 2022-08-04

**Authors:** Marcelo D. Catarino, Ana Rita Circuncisão, Bruno Neves, Catarina Marçal, Artur M. S. Silva, Maria Teresa Cruz, Susana M. Cardoso

**Affiliations:** 1 LAQV-REQUIMTE, Department of Chemistry, University of Aveiro, 3810-193 Aveiro, Portugal; 2 Department of Medical Sciences and Institute of Biomedicine—iBiMED, University of Aveiro, 3810-193 Aveiro, Portugal; 3 CNC.IBILI, Faculty of Pharmacy, University of Coimbra, Health Sciences Campus, 3000-548 Coimbra, Portugal

**Keywords:** brown macroalgae, *Himanthalia elongata*, marine bioactives, phenolic compounds, phlorotannins, gastrointestinal digestion, stability, anti-inflammatory, antioxidant

## Abstract

A phlorotannin extract was obtained from *Himanthalia elongata*, revealing a profile rich in fucophlorethol-type and carmalol-type compounds. When subjected to simulated gastrointestinal digestion, its levels of total phlorotannins and antioxidant activity, measured in vitro via NO^●^ and O_2_^●−^ scavenging assays, were reduced, thus suggesting that these compounds’ integrity and bioactivity are negatively affected by the digestive process. Nevertheless, when undigested vs. digested extracts were used on lipopolysaccharide-stimulated Raw 264.7 macrophages, both showed a strong inhibitory effect on the cellular NO^●^ production. In fact, although not statistically significant, the digested extract revealed a tendentially stronger effect compared to its undigested counterpart, suggesting that even though there is a decrease in the phlorotannins’ concentration after digestion, with a consequent loss of their scavenging properties, the possible degradation products being formed may exert their effects through the modulation of the intracellular signaling mechanisms. Overall, this study not only contributes to a better understanding of the phlorotannins’ composition of the species *H. elongata*, but also shows that, although the digestive process may affect the integrity and concentration of these compounds, this does not necessarily translate into loss of bioactivity, in particular the anti-inflammatory activity, probably owing to the bioactive effects that the degradation products of these phenolics may have at an intracellular level.

## 1. Introduction

Brown seaweeds are a well-known marine source of bioactive compounds, with a multitude of applications in numerous industrial fields. Among these, phlorotannins are drawing much attention, due to their potential health benefits and pharmacological applications [1]. These phenolic compounds, specific to brown seaweeds, are formed through the polymerization of several units of phloroglucinol, which can go from 126 Da to 650 kDa in molecular weight [2]. According to the type of linkages established between units, phlorotannins can be categorized as fucols (containing only C-C links), phlorethols (containing C-O-C links), fucophlorethols (containing both C-C and C-O-C links), and eckols (containing dibenzodioxin motifs) [3]. Phlorotannins with extra hydroxyl groups may also occur, and in these cases, they can be divided into fuhalols and carmalols, which are phlorethol-type and eckol-type phlorotannins, respectively, that contain at least one additional OH group in their structures [4].

These compounds are well-known antioxidants and anti-inflammatory agents, acting as scavengers of free radicals, or modulators of different cellular mechanisms involved in the inflammatory response [5]. However, most of the studies performed on phlorotannins’ bioactivities were carried out on pure compounds and/or phlorotannin-rich extracts, without taking into consideration the possible effects and alterations that gastrointestinal digestion may exert on these compounds before they reach their target.

Some steps have already been taken to address this shortcoming. Indeed, our previous studies have already shown how gastrointestinal digestion affects the stability and bioaccessibility of *Fucus vesiculosus’* phlorotannin-rich extracts, and how these compounds may modulate the gut microbiota’s growth and activity [6]. More recently, the concentrations of eckstolonol and dieckol, present in *Silvetia compresssa* hydroethanolic extract, were shown to progressively decrease during in vitro colonic fermentation with human fecal samples, suggesting that these compounds are being metabolized by the gut microbiota and transformed into other compounds [7]. In fact, according to the studies of Corona et al. [6], after the administration of capsules containing polyphenol extract from *Ascophyllum nodosum* to a group of 24 healthy volunteers, it was possible to detect the presence of molecular ions corresponding to hydroxytrifuhalol A, 7-hydroxyeckol and the C-O-C dimer of phloroglucinol in plasma and urine, as well as some conjugated forms (i.e., glucuronides and/or sulfates) with UV spectral characteristics relatable to phlorotannins. Nevertheless, information about the bioactive effects that phlorotannin-derived metabolites may exert is still quite limited. Our previous results from *F. vesiculosus’* phlorotannin-rich extracts demonstrated that the in chemico antioxidant activity of the digested extracts tended to decrease as the phlorotannins’ concentration decreased. However, the study of Corona et al. [6], carried out on human volunteers, reported alterations in the levels of plasma interleukin-8 after the intake of the *A. nodosum* polyphenol extract, indicating that either phlorotannins, in their free forms, are being sufficiently absorbed to exert biological effects, or the effects resulted from the metabolites formed during their digestion.

In this context, the aim of this work was to study the stability and anti-inflammatory activity of digested vs. non-digested phlorotannins from the species *Himanthalia elongata*, an edible brown seaweed that has a history of safe use and applications in multiple formulations (beef patties, restructured meats, low-fat frankfurters) [8,9,10,11], although poorly studied with regard to these type of compounds.

## 2. Materials and Methods

### 2.1. Extraction Procedure

The extraction was carried out as previously described by Catarino et al. [12]. Briefly, 30 g of dried *H. elongata* powder (Algamar, Pontevedra, Spain) was dispersed in 2100 mL of 70% acetone (Fisher, Pittsburgh, PA, USA), containing 1% glacial acetic acid (Fisher, Pittsburgh, PA, USA), and incubated at room temperature, under constant agitation. After 3 h, the mixture was filtered through cotton, to remove the solid residues, and then through a G4 glass filter. Afterwards, this crude extract (CRD) was concentrated in a rotary evaporator to approximately 250 mL. The concentrated CRD was defatted, using *n*-hexane (1:1, *v*/*v*; Fisher, Pittsburgh, PA, USA) several times—until a colorless non-polar fraction was obtained—and the aqueous phase was subjected to liquid–liquid extraction with ethyl acetate (1:1, *v*/*v*; Fisher, Pittsburgh, PA, USA), a further three times, to obtain a phlorotannin-purified fraction (EtOAc). Finally, the solvent was removed from the EtOAc fraction by rotary evaporation. Both CRD and EtOAc were then freeze dried and stored at −20 °C until further use.

### 2.2. Determination of Total Phlorotannin Content

The quantification of the total phlorotannin compounds (TPhC) was carried out using the 2,4-dimethoxybenzaldehyde (DMBA) colorimetric method, as described elsewhere [13]. For this method, equal volumes of DMBA (Sigma-Aldrich, St. Louis, MO, USA) stock solutions (2%, *w*/*v*) and HCl (6%, *v*/*v*), both prepared in glacial acetic acid, were mixed just before use (work solution). Afterwards, 250 µL of this solution was added to 50 µL of each extract in a 96-well plate and the reaction was incubated in the dark, at room temperature. After 60 min, the absorbance was read at 515 nm and the phlorotannin content was determined by using a regression equation of the phloroglucinol linear calibration curve (0.06–0.1 mg/mL). The results were expressed as mg phloroglucinol (Sigma-Aldrich, St. Louis, MO, USA) equivalents/g dry seaweed (mg PGE/g DS).

### 2.3. Ultra High Performance Liquid Chromatography-Diode Aarray Detector-Electropray Ionization/Mass Spectrometry Analysis

Chromatographic analysis of the phlorotannin-enriched EtOAc fraction was carried out as reported previously by Catarino et al. [12], using an Ultimate 3000 (Dionex Co., San Jose, CA, USA), an apparatus consisting of an autosampler/injector, a binary pump, a column compartment and an Ultimate 3000 diode array detector (Dionex Co., San Jose, CA, USA), coupled to a Thermo LTQ XL (Thermo Scientific, San Jose, CA, USA) ion trap mass spectrometer, equipped with an ESI source. The LC separation was carried out in a Hypersil Gold (ThermoScientific, San Jose, CA, USA) C_18_ column (100 mm length; 2.1 mm i.d.; 1.9 µm particle diameter, end-capped) maintained at 30 °C, and a binary solvent system composed of (A) acetonitrile (Fisher; Pittsburgh, PA, USA) and (B) 0.1% formic acid (*v*/*v*; Sigma-Aldrich, St. Louis, MO, USA). The solvent gradient started with 5–40% of solvent (A) over 14.72 min, from 40 to 100% over 1.91 min, then remaining at 100% for 2.19 min, before returning to the initial conditions. The flow rate was 0.2 mL/min and UV–Vis spectral data for all peaks were accumulated in the range: 200–700 nm, while the chromatographic profiles were recorded at 280 nm. Control and data acquisition of MS were carried out with the Thermo Xcalibur Qual Browser data system (ThermoScientific, San Jose, CA, USA). Nitrogen above 99% purity was used, and the gas pressure was 520 kPa (75 psi). The instrument was operated in negative mode, with the ESI needle voltage set at 5.00 kV and an ESI capillary temperature of 275 °C. The full scan covered the mass range from *m/z* 100 to 2000. CID–MS/MS experiments were performed for precursor ions, using helium as the collision gas, with a collision energy of 25–35 arbitrary units. All solvents used were of LC-MS grade.

### 2.4. Gastrointestinal Digestion Simulation

The simulation of the gastrointestinal digestion of the *H. elongata* sample extracts was performed following the method described by Campos et al. [14]. To simulate the oral digestion, 1 g of dried sample (CRD or EtOAc) was suspended in 20 mL of distilled water, followed by the adjustment of the pH to between 5.6 and 6.9, with 1 M NaHCO_3_ (Sigma-Aldrich, St. Louis, MO, USA), prior to the addition of 0.6 mL/min of α-amylase (Sigma-Aldrich, St. Louis, MO, USA) at 100 U/mL. Enzymatic digestion was carried out during 2 min of mastication, at 37 °C and 200 rpm. Before moving to the next compartment, the pH of the mouth digest was adjusted to 2.0, using 1 M HCl (Fisher, Pittsburgh, PA, USA), and then mixed with a simulated gastric juice consisting of 25 mg/mL of pepsin (Sigma-Aldrich, St. Louis, MO, USA) added at a ratio of 0.05 mL/mL of mouth digest. Incubation was carried out over 60 min at 37 °C and 130 rpm. Finally, for intestinal digestion the pH of gastric digest was adjusted to 6.0, using 1 M NaHCO_3_, prior to the addition of a simulated intestinal juice consisting of 2 g/L of pancreatin (Sigma-Aldrich, St. Louis, MO, USA) and 12 g/L bile salts (Sigma-Aldrich, St. Louis, MO, USA) at a ratio of 0.25 mL/mL of gastric digest. The samples were then incubated for 120 min, at 37 °C and 45 rpm, to mimic a long intestinal-digestion process.

### 2.5. Radical Scavenging Experiments

#### 2.5.1. Superoxide Anion (O_2_^●−^) Scavenging Assay

Following the O_2_^●−^ scavenging assay described by Pereira et al. [15], 75 µL serial dilutions of *H. elongata* samples were mixed with: 100 µL of 300 µM β-NADH (Sigma-Aldrich, St. Louis, MO, USA); 75 µL of 200 µM NBT (Sigma-Aldrich, St. Louis, MO, USA); and 50 µL of 15 µM PMS (Sigma-Aldrich, St. Louis, MO, USA), in a 96-well plate. After 5 min, the absorbances at 560 nm were recorded; the inhibition was calculated as the concentration capable of scavenging 50% of O_2_^●−^ (IC_50_). Gallic acid (Sigma-Aldrich, St. Louis, MO, USA) was used as the reference compound.

#### 2.5.2. Nitric Oxide (NO^●^) Scavenging Assay

The NO^●^ scavenging method was adapted from Catarino et al. [16]. Briefly, 100 µL of serial dilutions of *H. elongata* samples were mixed with 100 µL of sodium nitroprusside (3.33 mM in 100 mM sodium phosphate buffer, pH 7.4; Acros Organics, Hampton, NH, USA) and incubated for 15 min under a fluorescent lamp (Tryun 26 W). Next, 100 µL of Griess reagent (consisting of 0.5% sulfanilamide (Acros Organics, Hampton, NH, USA) and 0.05% N-(1-naphthyl)-ethylenediamine dihydrochloride (VWR, Radnor, PA, USA) in 2.5% H_3_PO_4_) was added to the mixture, which was incubated for another 10 min at RT, in the dark. The absorbance was then measured at 562 nm, and the NO^●^ scavenging capacity was calculated as the concentration of sample capable of scavenging 50% of the radical. Ascorbic acid (Sigma-Aldrich, St. Louis, MO, USA) was used as the reference compound.

### 2.6. Cell Experiments

#### 2.6.1. Cell Culture

Raw 264.7 cells—a mouse leukemic monocyte macrophage cell line (ATCC TIB-71)—were cultured (as described elsewhere [17]) in Dulbecco’s modified Eagle’s medium (DMEM; Sigma-Aldrich, St. Louis, MO, USA), supplemented with: 10% inactivated fetal bovine serum (Gibco, Paisley, UK); 100 U/mL penicillin (Sigma-Aldrich, St. Louis, MO, USA); and 100 µg/mL streptomycin (Sigma-Aldrich, St. Louis, MO, USA), at 37 °C in a humidified atmosphere of 95% air and 5% CO_2_. Throughout the experiments, cells were monitored by microscopy in order to detect any morphological change.

#### 2.6.2. Assessment of Cell Viability

The effect of each sample on cell viability/metabolic activity was evaluated according to the resazurin assay adapted from previous works [18]. For this assay, cells (6 × 10^4^ cells/well) were plated in 96-well plates and allowed to stabilize overnight. Cells were then exposed to serial dilutions of each sample reconstituted in DMEM with 0.5% dimethyl sulfoxide (DMSO; Fisher, Pittsburgh, PA, USA), which has previously been shown to have minimal impact on Raw 264.7 viability [19]. After 24 h incubation, 50 µM resazurin (Sigma-Aldrich, St. Louis, MO, USA) was added to the cells, 3 h prior to recording absorbance at 570 nm, with a reference wavelength of 620 nm, using a standard spectrophotometer. The results were expressed relative to untreated cells viability/metabolic capacity.

#### 2.6.3. Inhibition of Lipopolysaccharide (LPS)-Stimulated NO^●^ Production

The effect of CRD, EtOAc and corresponding intestinal-digested samples of *H. elongata* on the nitrite production in LPS-stimulated Raw 264.7 cells was measured using a colorimetric reaction, with Griess reagent, as described elsewhere [20]. For this, cells were plated and treated with the *H. elongata* samples, as described above in Section 2.6.2. After 1 h of sample exposure, 50 ng/mL of LPS (from Escherichia coli—serotype 026:B6; Sigma-Aldrich, St. Louis, MO, USA) was added to each well (except for the negative control) and allowed to incubate for 24 h. The cell-free supernatants were then collected and diluted with equal volumes of Griess reagent for 30 min, in the dark, prior to the absorbance recording at 550 nm. The percentage of nitrite production was calculated based on the LPS control, which was considered to represent 100%.

### 2.7. Statistical Analysis

Data were expressed as mean ± standard deviation (SD) of three similar and independent experiments in the scavenging experiments, whereas for the cellular experiments, data were expressed as mean ± standard error of the mean (SEM) of three similar and independent experiments. One-way ANOVA, followed by Tukey’s post-hoc test, was performed for the scavenging assays, while one-way ANOVA, followed by Dunnet’s post-hoc test, was performed for the experiments carried out on cells. The statistical tests were applied using GraphPad Prism, version 7.00 (GraphPad Software, San Diego, CA, California) and the significance level was *p* < 0.05.

## 3. Results

### 3.1. Total Phlorotannin Content of the H. elongata Extract and Respective Fractions

The 70% acetone extract of *H. elongata* represented approximately 33% of the dried algal material and had a total phlorotannin content of 0.15 ± 0.02 phloroglucinol equivalents (PGE)g/100 g (Table 1). Compared with the work developed by Ferraces-Casais et al. [21] who performed the extraction of *H. elongata* using a mixture of methanol–hexane–dichloromethane (2:1:1), the TPhC obtained herein is slightly higher, although higher TPhC values (1.4–20.7 g/100 g) have been described for crude extracts of this species obtained with other solvents, including methanol 20–100% or ethanol 80% [22,23,24,25,26].

To remove other co-extracted components and obtain an extract with higher concentration of phlorotannins, a liquid–liquid partitioning was carried out using solvents of different polarities. A total of three fractions resulted from this liquid–liquid partitioning, namely the *n*-hexane fraction (HEX), the ethyl acetate fraction (EtOAc) and the aqueous residue (AQ). Of these, the EtOAc was the fraction with the highest TPhC (2.15 ± 0.09 g PGE/100 g), which is coherent with previous authors, who have also found that phlorotannins are retained in the highest quantities in the ethyl acetate phase [12,27,28].

### 3.2. Characterization of Phlorotannin-Rich Fraction

**Figure 1:**
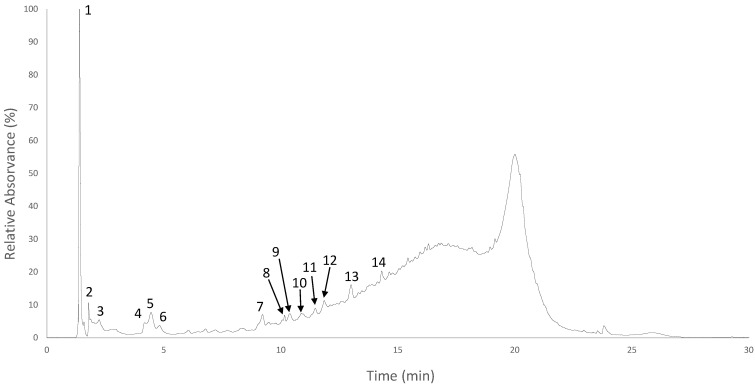
Chromatographic profile of the ethyl acetate fraction of *H. elongata* extract at 280 nm. Peaks marked with numbers correspond to the tentatively assigned compounds listed in Table 2.

Due to its higher TPhC, EtOAc was, furthermore, taken for UHPLC-ESI-MS/MS analysis, to proceed with the elucidation of its phlorotannin profile. Figure 1 shows the UV chromatograms recorded at 280 nm. Overall, a total of 34 compounds were detected, with molecular weights ranging from 3 to 20 phloroglucinol units.

Fucophlorethol-type compounds, with a diverse degree of polymerization, were the most frequently appearing group. These were [M–H]^−^ at *m/z* 373 (DP3, at 4.9 min), [M–H]^—^ at *m/z* 869 (DP7, at 13.0 min), [M–H]^−^ at *m/z* 993 (DP8, 3 isomers at 10.2, 10.4 and 13.0 min), [M–H]^−^ at *m/z* 1117 (DP9, 3 isomers at 10.2, 10.9 and 11.5 min), [M–H]^−^ at *m/z* 1241 (DP10, 5 isomers at 10.2, 10.4, 10.9, 11.5 and 11.9 min), [M–H]^−^ at *m/z* 1365 (DP11, 2 isomers at 10.9 and 11.5 min) and [M–H]^−^ at *m/z* at 1489 (DP12, 3 isomers at 11.5, 11.9 and 13.0 min), all showing typical phlorotannin fragments (e.g., *m/z* at 229, 245, 247, 249, 353, 369, 371, 373, 477 and so on [29]) along with common losses of one or more phloroglucinol or *O*-phloroglucinol units (124/126 and 142 Da, respectively), as well as losses that indicate cross-ring cleavages such as −28, −44, −72, −84 or −98 (and their combination with phloroglucinol and/or water units), which is a typical fragmentation pattern of fucophlorethols [12]. Another isomer of a compound with [M–H]^−^ at *m/z* 869 eluting at 9.2 was identified as octophlorethol, due to the absence of fragment ions indicating cross-ring cleavages and the occurrence of several fragment ions indicating the presence of C-O-C linkages, including [M–H]^−^ at *m/z* 727 (-*O*-phloroglucinol), *m/z* 603 (-bifuhalol) and *m/z* 477 (-trifuhalol) [12].

After fucophlorethols, phlorethohydroxycarmalols appeared as the second most abundant group of compounds detected in this sample. Based on the literature, these were tentatively assigned to pentaphlorethohydroxycarmalol ([M–H]^−^ at *m/z* 883, peak 8), triphlorethodihydroxycarmalol ([M–H]^−^ at *m/z* 635, peak 9), hexaphlorethohydroxycarmalol ([M–H]^−^ at *m/z* 1007, peak 10) and heptaphlorethohydroxycarmalol ([M–H]^−^ at *m/z* 1131, peak 11), all demonstrating a fuhalol-like fragmentation pattern, i.e., showing common losses that correspond to *O*-phloroglucinol moieties and its further combination with more phloroglucinol units and water (e.g., −142, −266, −390, −406 or 530 Da) [30]. Interestingly, carmalols are a group of phlorotannins that are usually related to the species *Ishigue okamurae* [31,32,33,34], although they have already been described in the species *Sargassum fusiforme* [30] and *Carpophyllum maschalocarpum* [35]. However, to the authors knowledge, no previous studies reported this family of phlorotannins in *H. elongata* before.

Several compounds co-eluting at 14.4 min showed deprotonated molecular ions at *m/z* 868, 930, 992, 1054, 1178 and 1240, which are consistent with the doubly charged ions of phlorotannins with DP 14, 15, 16, 17, 19 and 20, respectively [36], although their signals were too weak to run a proper MS/MS analysis.

Notably, although a full structural elucidation was not achieved, some other compounds have been tentatively identified as phlorotannin derivatives ([M–H]^−^ at *m/z* 267, at 4.2 min, [M–H]^−^ at *m/z* 419, 2 isomers at 4.5 and 4.9 min, and [M–H]^−^ at *m/z* 1189, at 10.9 min), on the basis of their MS/MS spectra, which exhibited some fragments (e.g., *m/z* at 245, 249, 373) and/or neutral losses (e.g., −44, −126, −248) that are indicative of phlorotannin compounds [37].

### 3.3. Effects of Gastrointestinal Digestion on the Stability and Antioxidant Activity H. elongata Phlorotannin-Rich Extracts

**Figure 2:**
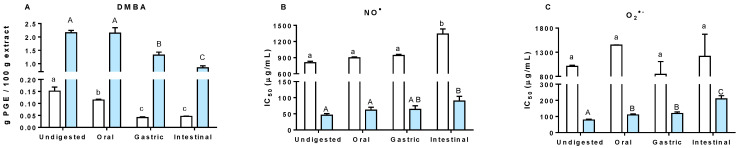
Effect of gastrointestinal digestion on the total phlorotannin content (**A**) NO^●^ radical scavenging (**B**) and O_2_^●−^ radical scavenging (**C**) activities of CRD (white bars) and EtOAc (blue bars) extracts from *H. elongata*. Different letters (a–c) indicate significant differences between means of the white bars (*p* < 0.05). Different capital letters (A–C) indicate significant differences between means of the blue bars (*p* < 0.05).

To evaluate the stability of *H. elongata* phlorotannins throughout the digestive tract, both CRD and EtOAc were submitted to a simulated gastrointestinal (GIT) digestion, tracking a quantification of the total phlorotannin content after each gut compartment. At the beginning of the procedure, 1 g of each extract was loaded into the system, corresponding to 0.15 ± 0.017 and 2.15 ± 0.093 g PGE/100 g of CRD and EtOAc, respectively. However, as shown in Figure 2A, the TPhC of both samples gradually decreased after each step of the digestive system. At the end of the intestinal phase, the observed TPhC accounted for 0.05 ± 0.001 and 0.84 ± 0.075 µg/mL (for CRD and EtOAc, respectively), corresponding to approximately 33 and 39% of the initial TPhC, respectively. This was an expected result, since the transformation and metabolization of phenolic compounds in general is a phenomenon that is well documented in the literature [38]. In fact, these observations are in line with our previous work on phlorotannin-rich extracts from *Fucus vesiculosus* in which the TPhC was also found to progressively decrease as they moved forward in the digestive process [39].

The decreasing concentrations of phlorotannins in the samples was reflected in the antioxidant activity of the extracts. This is particularly true for the EtOAc, since in both NO^●^ and O_2_^●−^ experiments, a progressive increase of the IC_50_ values, and therefore, decrease of antioxidant activity, was found after each step of the digestive process, correlating well with the TPhC of the samples at the corresponding digestive stage (R^2^ = −0.88 and −0.89 for NO^●^ and O_2_^●−^, respectively). The results obtained for CRD, however, did not follow the same linearity. For the NO^●^ experiment, the loss of activity was found only in the last stage of gastrointestinal digestion (intestinal digestion) while for the O_2_^●−^ experiment, the alterations did not reveal statistical significance. In this case, it should be considered that the non-digested CRD demonstrates a significantly lower TPhC when compared to EtOAc, which translates into a considerably low antioxidant activity right from the start.

### 3.4. Effects of Gastrointestinal Digestion on Anti-Inflammatory Properties of H. elongata Phlorotannin-Rich Extracts

**Figure 3:**
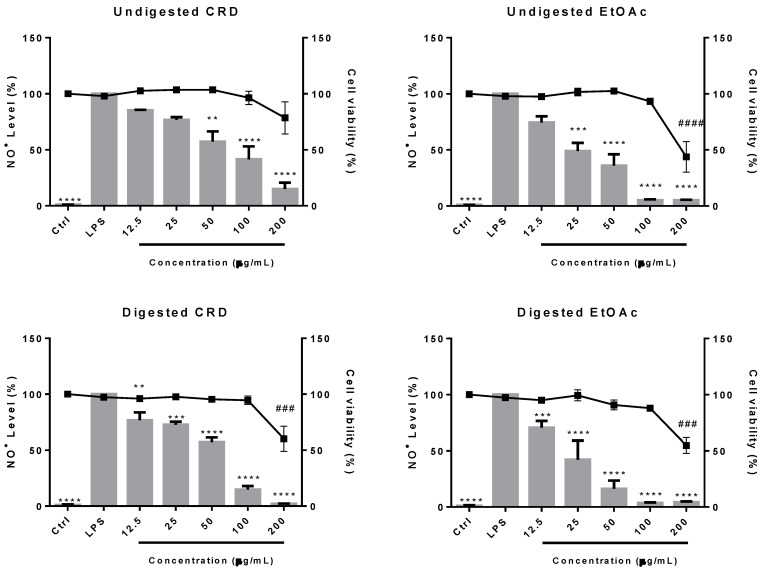
Effects of *H. elongata* crude extract (CRD) and ethyl acetate fraction (EtOAc) on the NO^•^ production (grey bars) and viability (■) of LPS-stimulated Raw 264.7 cells. Data represent the mean ± SEM from at least 3 independent experiments. ** *p* < 0.01, *** *p* < 0.001 and **** *p* < 0.0001, indicate that NO^•^ production is significantly different from the positive control (with LPS), and ### *p* < 0.001 and #### *p* < 0.0001 indicate that cell viability is statistically different from the negative control (CTRL, without LPS), as determined by one-way ANOVA, followed by Dunnet’s post-hoc test.

To study the impact of gastrointestinal digestion on the bioactive properties of *H. elongata* phlorotannin extracts, the next step was to evaluate their anti-inflammatory activities in a biological system, i.e., in Raw 264.7 cells stimulated with the Toll-like receptor 4 (TLR-4) agonist, lipopolysaccharide (LPS). For that, cells were treated with different concentrations of non-digested or fully-digested extracts (i.e., after undergoing the three digestive phases), and the results are depicted in Figure 3. As expected, under normal conditions, i.e., in the absence of LPS, the production of NO^●^ was almost negligible, while a drastic increase was observed after the addition of LPS, denoting the activation of the macrophages’ inflammatory response. The addition of the *H. elongata* phlorotannin extracts to the medium resulted in a dose-dependent inhibition of the NO^●^, produced by the LPS-stimulated cells. Notably, at 100 µg/mL, non-digested CRD and EtOAc were able to reduce the LPS-induced NO^●^ release by 70 and 94.8%, respectively, while their digested counterparts caused a reduction of 85.2 and 96.4%, respectively, in the same radical. At 200 µg/mL a decrease in cell viability, to below 80%, was noted in the four samples, indicating a possible cytotoxic effect.

Overall, comparing both samples, EtOAc displayed consistently better results, either for non-digested (5.2 ± 0.7 versus 41.5 ± 11.5%, for EtOAc and CRD respectively, *p* < 0.5, unpaired *t*-test) or digested samples (3.6 ± 0.5 versus 14.8 ± 3.2%, for EtOAc and CRD respectively, *p* < 0.5, unpaired *t*-test), matching the tendency previously observed in chemico. Nevertheless, in the light of the DMBA results previously described, it is worth noting how effective CRD samples also were at inhibiting the LPS-induced NO^●^ release, indicating that other non-phlorotannin compounds present in the extract might be contributing to the results obtained. Moreover, when comparing the non-digested with the digested samples, it was interesting to notice a tendential (although not statistically significant) increase of activity for the latter. This was particularly visible for the concentration of 100 µg/mL in CRD samples (41.5 ± 11.5 versus 14.8 ± 3.2% for non-digested and digested CRD, respectively) and 50 µg/mL in EtOAc samples (36.0 ± 10.0 versus 16.4 ± 7.2% for non-digested and digested EtOAc, respectively). Considering the significantly lower TPhC of the digested extracts compared with their non-digested counterparts, as observed in Section 3.3., a reduction of the anti-inflammatory capacity of the digested extracts, measured as the capacity to inhibit the production of NO^•^ in LPS-stimulated macrophages, was expected, especially in the EtOAc extract. One possible explanation for these observations is that the gastrointestinal digestion might be promoting the breakdown of complex phlorotannins’ structures and/or the formation of certain metabolites that might exert stronger inhibitory effects on the NO^•^ production/release. Indeed, previous studies on *F. vesiculosus* phlorotannins have shown a possible relation between phlorotannins’ complexity and their anti-inflammatory effects towards inhibition of NO^•^ production by LPS-exposed Raw 264.7 macrophages, with fractions of lower molecular weight phlorotannins showing better results when compared with the fractions of high molecular weight phlorotannins [20].

## 4. Conclusions

In this study, the phlorotannin profile of *H. elongata*, which was quite underexplored until now, was shown to contain several compounds with polymerization degrees ranging from 3 to 20 phloroglucinol units, mostly belonging to the fucophlorethol group, but also with a significant contribution of carmalols. Moreover, this work represents an important contribution to the understanding of the effects of gastrointestinal digestion on the stability and bioactive properties of these brown seaweed-derived phenolics, showing that, although the concentrations of phlorotannins as well as their scavenging properties may significantly decrease after digestion, it does not necessarily translate into the loss of biological activity, since the results showed that the digested extract exerted very similar (even tendentially higher) anti-inflammatory properties on LPS-stimulated Raw 264.7 macrophages, compared to the non-digested extract. Based on these results, it is possible to infer that, although the digestive process may contribute to the breakdown of phlorotannins, the resultant degradation products may be relevant players in the health benefits attributed to the consumption of these compounds.

## Figures and Tables

**Table 1:** Extraction yield (as % *w*/*w* of algal powder for crude extract and % *w*/*w* of crude extract for the fractions) and total phlorotannin content of *H. elongata* crude extract and further fractions.

Sample	Yield (%)	TPhC (g PGE/100 g ext)
CRD	32.9 ± 0.49	0.15 ± 0.02
HEX	3.68 ± 0.08	0.28 ± 0.04
EtOAc	3.39 ± 0.32	2.15 ± 0.09
AQ	82.72 ± 0.62	0.05 ± 0.002

CRD: Crude extract; HEX: Hexane fraction; EtOAc: Ethyl acetate fraction; AQ: Aqueous residue; TPhC: Total phlorotannin content.

**Table 2:** Assignment of the compounds present in the ethyl acetate fraction of *H. elongata* extract analyzed by UHPLC-ESI-MS/MS.

Peak	RT (min)	[M–H]^−^ (*m/z*)	MS/MS Ions	Tentative Assignment
1	1.4	191	111, 173, 129,147, 87	Citric acid
		391	217, 373, 191, 111, 259, 155, 173	Citric acid derivative
2	1.8	391	217, 3 73, 191, 111	Citric acid derivative
3	2.2	209	163, 165, 137, 181, 191, 133, 93, 173	Unkown
4	4.2	267	221, 223, 179, 249	Phlorotannin derivative
5	4.5	419	401, 375, 329, 331, 373, 383, 347, 387, 357, 307, 279, 245	PT derivative
6	4.9	419	375, 401, 329, 331, 373, 383, 293, 347, 321, 307	PT derivative
		373	233, 247, 229, 125, 189, 329, 355, 289	Fucophlorethol
7	9.2	869	851, 727, 603, 745, 619, 495, 369, 477, 353	Octophorethol
8	10.2	883	865, 847, 741, 459, 477, 617, 353, 261	Pentaphlorethohydroxycarmalol
		993	975, 869, 851, 603, 745, 921, 621, 789, 461, 371	Fucophlorethol 8 U
		1117	1099, 1045, 992, 1055, 1089, 973, 921, 851, 603, 581, 495, 443, 415	Fucophlorethol 9 U
		1241	1223, 1169, 1179, 1099, 993, 869, 1027, 495, 457	Fucophlorethol 10 U
9	10.4	635	617, 509, 493, 369, 385, 245, 229	Triphlorethodihydroxycarmalol
		993	975, 869, 851, 931, 727, 601, 743, 477, 353	Fucophlorethol 8 U
		1241	1223, 1169, 1179, 1099, 975, 993, 1053, 867, 851, 745,	Fucophlorethol 10 U
10	10.9	1007	989, 971, 865, 781, 963, 883, 443,	Hexaphlorethohydroxycarmalol
		1117	1099, 973, 869, 1035, 443, 743, 337, 477	Fucophlorethol 9 U
		1189	1171, 1145, 1063, 975, 941, 895, 773,	PT derivative
		1241	1223, 1179, 1169, 1117, 993, 1161, 1099,975, 869, 743, 691, 475	Fucophlorethol 10 U
		1365	1347, 1293, 1117, 1239, 975, 1223, 1169, 867, 727, 603	Fucophlorethol 11 U
		1489	1471, 1417, 1291, 1365, 1099, 973, 821, 869, 759, 495	Fucophlorethol 12 U
11	11.5	1117	1099, 867, 993, 851, 1054, 975, 743, 727, 603, 477, 443, 351	Fucophlorethol 9 U
		1131	1113, 1095, 1005, 883, 989, 865, 759, 721, 659, 585, 475, 449	Heptaphlorethohydroxycarmalol
		1241	1223, 1169, 1117, 993, 1159, 1099, 867, 851, 727, 743, 477	Fucophlorethol 10 U
		1365	1347, 1301, 1293, 1241, 1117, 1169, 991, 1099, 849, 725, 585	Fucophlorethol 11 U
		1489	1471, 1427, 1241, 1033, 727, 495, 661, 869, 991, 1115, 1223, 1355	Fucophlorethol 12 U
12	11.9	1241	1223, 1117, 1179, 993, 1099, 1445, 869, 727, 975, 849, 475, 443, 585	Fucophlorethol 10 U
		1489	1471, 1365, 1427, 1417, 1445, 1337, 1115, 849, 727, 517, 435	Fucophlorethol 12 U
13	13.0	869	859, 851, 797, 735, 745, 807, 847, 787, 753, 681, 727, 619, 353, 609	Fucophlorethol 7 U
		993	975, 931, 921, 983, 869, 913, 851, 813, 797, 743, 601, 621, 451, 475, 371	Fucophlorethol 8 U
		1489	1471, 1417, 1347, 1293, 1161, 1117, 1363, 1217, 831, 551, 989	Fucophlorethol 12 U
14	14.4	868.5	Low MS signal	Phlorotannin 14 U
		930.5	Low MS signal	Phlorotannin 15 U
		992.5	Low MS signal	Phlorotannin 16 U
		1054.5	Low MS signal	Phlorotannin 17 U
		1178.5	Low MS signal	Phlorotannin 19 U
		1240.5	Low MS signal	Phlorotannin 20 U

RT: Retention time; MS: Mass spectrometry; U: units.

## Data Availability

The data presented in this study are available in the article.
